# Capillaroscopic patterns are associated with interstitial lung disease, skin fibrosis, anti-Th/To antibodies and quality of life in systemic sclerosis: a prospective cross-sectional study

**DOI:** 10.3389/fmed.2026.1808581

**Published:** 2026-04-15

**Authors:** Jakub Trefler, Anna Pasierb, Lidia Lech, Hubert Czaplicki, Katarzyna Życińska

**Affiliations:** 1 Department of Rheumatology, Connective Tissue Diseases and Rare Diseases, National Medical Institute of the Ministry of the Interior and Administration, Warsaw, Poland; 2 Department of Family Medicine, Medical University of Warsaw, Warsaw, Poland

**Keywords:** biomarkers, capillaroscopy, interstitial lung disease, patient-reported outcome measures, systemic sclerosis

## Abstract

**Background:**

Capillaroscopic patterns in systemic sclerosis (SSc) are routinely assessed by nailfold videocapillaroscopy (NVC), yet their broader clinical relevance remains incompletely defined.

**Methods:**

In a prospective cross-sectional study of 70 SSc patients, we evaluated associations between NVC patterns and key clinical domains: interstitial lung disease (ILD), skin fibrosis (the modified Rodnan Skin Score, mRSS), SSc-specific autoantibody profile including anti-Th/To, immunosuppressive treatments, and patient-reported outcome measures (PROMs): SSc Quality of Life scale (SScQoL), Health Assessment Questionnaire-Disability Index (HAQ-DI), and Visual Analog Scales (VAS) for pain and disease activity (PtGA). Statistical methods included Spearman correlation, Mann–Whitney *U*-test, receiver operating characteristic (ROC) analysis, and logistic regression. *p*-values were adjusted using the false discovery rate (FDR).

**Results:**

Advanced NVC patterns (4–5) were significantly associated with ILD (*OR* = 3.73, 95% *CI*: 1.42–9.81, *q* = 0.0058), and this association remained consistent across multiple multivariable and parsimonious logistic regression models adjusting for anti-Scl-70 status, disease duration, or skin fibrosis (mRSS), with effect estimates around fourfold increased odds of ILD. ROC analysis demonstrated diagnostic discrimination for ILD based on NVC pattern (*AUC* = 0.688). Higher NVC severity was also associated with greater skin fibrosis (ρ = +0.38, *q* = 0.0283), with progressive increases in median mRSS from NVC pattern 2 to 5 (4.0 → 11.0). Strong associations were found with PROMs: worse SScQoL (*q* = 0.0040), higher HAQ-DI (*q* = 0.0027), and elevated PtGA (*q* = 0.0040). A novel inverse correlation was identified between NVC patterns and anti-Th/To antibody positivity (*q* = 0.022). Exposure to cyclophosphamide (CYC) or mycophenolate mofetil (MMF) was associated with more severe capillaroscopic damage (both *q* = 0.019) most likely reflecting greater disease severity rather than a treatment effect.

**Conclusion:**

Our study demonstrates significant associations between NVC severity and selected clinical, serological, therapeutic, and patient-reported domains in SSc.

## Methods

### Study design and participants

This was a prospective, cross-sectional, single-center study conducted at a tertiary rheumatology and rare diseases center. Seventy adult patients with SSc were consecutively enrolled between February 2024 and December 2025, all meeting the 2013 ACR/EULAR classification criteria (1). Patients with sine scleroderma or localized forms (e.g., morphea) were excluded. Other exclusions were limited to significant renal impairment (creatinine clearance < 45 ml/min), advanced cardiac disease (NYHA class III–IV), or clinically relevant hepatic insufficiency. No formal sample size calculation was performed; all eligible patients during the recruitment period were included.

The study received institutional ethics approval, and all participants provided informed consent. Clinical, laboratory, capillaroscopic, and PROM data were collected during a single, short (1–4 day) inpatient stay, typically scheduled for disease evaluation, treatment review, or rheological therapy with iloprost.

### Nailfold videocapillaroscopy

All NVC examinations were performed by the same physician with seven years of specialty training in rheumatology and dermatology and formal EULAR certification in capillaroscopy. The procedure was conducted following standardized EULAR Study Group on Microcirculation in Rheumatic Diseases' protocols. NVC was performed using magnifications of 50 × (for panoramic assessment) and 200 × (for detailed evaluation). Eight fingers (digits 2–5 of both hands) were examined in each patient. For quantitative assessment, three adjacent 1-mm fields were analyzed at 200 × magnification (total 3 mm per finger), and three images were recorded per finger. Images were categorized into one of six defined patterns:

1. Normal pattern
2. Non-specific abnormalities
3. Early scleroderma pattern
4. Active scleroderma pattern
5. Late scleroderma pattern
6. Scleroderma-like pattern

Although all six categories were available in the classification framework, only patterns 2 through 5 were observed in this cohort. Patterns 1 (normal) and 6 (scleroderma-like) did not occur in any patient, but were not excluded *a priori* and were fully eligible. This reflects the presence of established microangiopathy in a clinically confirmed SSc cohort rather than any capillaroscopy-based inclusion or exclusion criterion. NVC patterns were further used as both ordinal and binary variables in statistical analyses (e.g., 2–3 vs. 4–5 severity grouping). This dual approach was chosen to balance statistical sensitivity and clinical interpretability: ordinal scaling (2–5) preserves the full spectrum of capillaroscopic severity for correlation analyses, while binary grouping (2–3 vs. 4–5) reflects a clinically validated transition in microvascular pathology. Specifically, patterns 2 and 3 (non-specific abnormalities and early scleroderma pattern) are characterized by preserved capillary density (≥7/mm), minimal architectural disarray, and absence or rarity of neoangiogenesis or dropouts—hallmarks of microvascular integrity. In contrast, patterns 4 and 5 (active and late scleroderma patterns) exhibit progressive capillary loss (< 6/mm), frequent giant capillaries and hemorrhages, significant architectural disorganization, and, in late stages, compensatory neoangiogenesis. This dichotomy aligns with the established progression of scleroderma microangiopathy, as defined by Cutolo et al. and the EULAR Study Group (2–4), and provides a robust pathophysiological rationale for categorical modeling of disease severity. Additionally, binary grouping mitigates type I/II error risks associated with small sample sizes in extreme categories.

### Patient-reported outcome measures (PROMs)

Four validated PROM instruments were administered, all during the same inpatient visit and supervised by trained personnel blinded to clinical and laboratory data:

1. Systemic Sclerosis Quality of Life Questionnaire (SScQoL): A disease-specific tool consisting of 29 dichotomous (true/false) items (range: 0–29); higher scores reflect worse health-related quality of life (HRQoL) (5, 6).
2. Health Assessment Questionnaire—Disability Index (HAQ-DI): Evaluates functional disability across eight domains; range: 0–3, with higher scores indicating greater disability (7–9).
3. Visual Analog Scale (VAS) for Pain: A 100-mm horizontal line representing patient self-assessed pain severity over the past week; higher values reflect more intense pain (10, 11).
4. Visual Analog Scale for Patient Global Assessment of Disease Activity (PtGA): A 100-mm scale measuring the patient's overall perception of disease activity; higher scores indicate greater perceived disease burden (12–15).

These PROMs were selected based on psychometric validation in SSc populations and availability of Polish language versions. Other SSc-specific instruments (e.g., ScleroID) could not be included due to lack of access to validated Polish versions or licensing constraints at the time of study design.

### Study variables

A total of 36 variables (Table 1) were collected across seven domains and used for descriptive, correlative, and regression analyses:

1. Demographic and socioeconomic variables (6): Including sex, age, education level, place of residence, family status, and employment.
2. Capillaroscopic pattern variables (6): NVC patterns (1–6), ordinal severity (patterns 2–5), and binary groupings (2–3 vs. 4–5), as detailed in the NVC section.
3. Patient-reported outcomes (4): Total scores from SScQoL, HAQ-DI, VAS pain, and PtGA.
4. Clinical features (5): Including disease duration, presence of ILD confirmed by high-resolution computed tomography (HRCT) (16), pulmonary arterial hypertension, gastrointestinal involvement (documented reflux, dysphagia, malabsorption and or results of imaging tests), and mRSS (17).
5. Laboratory markers (5): Inflammatory and renal parameters: ESR, CRP (18, 19), IL-6, creatinine, and eGFR (20).
6. Immunological parameters (5): ACA, Scl-70, anti-Th/To, complement C3c, and C4 (21, 22).
7. Treatment exposure (5): Current or past use of MMF, MTX, AZA, and CYC (historical only), and ongoing iloprost. Dose, duration, and cumulative treatment exposure were not available.

**Table 1:** Cohort characteristics (*N* = 70).

Variable	Value
1. Demographic and socioeconomic variables	
Sex	*n* = 70 | female: 58 (82.9%), male: 12 (17.1%)
Age (years)	*n* = 70 | 55.0 (47.0–68.0)
Education	*n* = 68 | higher: 25 (36.8%), other: 43 (63.2%)
Place of residence	*n* = 68 | urban: 52 (76.5%), rural: 16 (23.5%)
Family status (children/none)	*n* = 68 | with children: 42 (61.8%), without: 26 (38.2%)
Employment status	*n* = 68 | employed: 34 (50.0%), other: 34 (50.0%)
2. Capillaroscopic patterns	
Pattern 1 (normal)	*n* = 70 | 0 (0.0%)
Pattern 6 (scleroderma-like)	*n* = 70 | 0 (0.0%)
Pattern 2 (non-specific abnormalities)	*n* = 70 | 10 (14.3%)
Pattern 3 (early scleroderma)	*n* = 70 | 34 (48.6%)
Pattern 4 (active scleroderma)	*n* = 70 | 19 (27.1%)
Pattern 5 (late scleroderma)	*n* = 70 | 7 (10.0%)
Binary NVC patterns (2–3 vs. 4–5)	*n* = 70 | 2–3: 62.9%, 4–5: 37.1%
Binary NVC patterns (2 vs. 3–5)	*n* = 70 | 2: 14.3%, 3–5: 85.7%
3. Patient-reported outcome measures (PROs)	
SScQoL	*n* = 70 | 13.5 (9.0–20.0)
HAQ-DI	*n* = 70 | 0.8 (0.2–1.2)
VAS pain	*n* = 70 | 50.0 (25.8–72.2)
PtGA	*n* = 70 | 51.5 (32.8–67.5)
4. Clinical features	
Disease duration (years)	*n* = 70 | 3.0 (1.0–9.0)
Interstitial lung disease (ILD)	*n* = 69 | 24 (34.3%)
Pulmonary arterial hypertension(PAH)	*n* = 69 | 5 (7.1%)
Gastrointestinal involvement	*n* = 69 | 15 (21.4%)
Modified Rodnan skin score(mRSS)	*n* = 67 | 5.0 (2.0–8.5)
5. Laboratory markers (inflammatory and renal)	
Erythrocyte sedimentation rate (ESR)	*n* = 69 | 14.0 (8.0–26.0)
C-reactive protein (CRP)	*n* = 69 | 1.9 (0.8–3.9)
Interleukin-6 (IL-6)	*n* = 66 | 3.8 (2.1–6.9)
Creatinine	*n* = 69 | 0.7 (0.7–0.9)
Estimated glomerular filtration rate (eGFR)	*n* = 69 | 95.0 (83.0–107.0)
6. Immunological parameters	
Anti-centromere antibody (ACA) positive	*n* = 66 | 34 (51.5%)
Scl-70 (anti-topoisomerase I) positive	*n* = 66 | 32 (48.5%)
Anti-Th/To positive	*n* = 63 | 18 (28.6%)
Complement C3c	*n* = 66 | 114.6 (102.0–126.7)
Complement C4	*n* = 66 | 24.8 (17.7–28.8)
7. Treatment variables	
Mycophenolate mofetil (MMF)	*n* = 70 | 30 (42.9%)
Methotrexate (MTX)	*n* = 70 | 29 (41.4%)
Cyclophosphamide (CYC)	*n* = 70 | 6 (8.6%)
Azathioprine (AZA)	*n* = 70 | 3 (4.3%)
Intravenous iloprost	*n* = 70 | 44 (62.9%)

### Bias minimization and data collection protocol

To minimize bias and ensure methodological consistency:

- Patients were enrolled consecutively to avoid selection bias.
- All NVC examinations were conducted by a single EULAR-certified physician to reduce inter-observer variability.
- Clinical assessments, including mRSS, were performed by trained rheumatologists calibrated to EULAR standards prior to study initiation.
- All PROMs were administered by staff specifically trained in PROM instruments and blinded to clinical and laboratory data.
- Centralized and standardized laboratory assays were used for inflammatory and immunological markers.
- Double data entry with cross-checking was performed for consistency and completeness.
- All study procedures were scheduled on the same day to control for temporal variation between capillaroscopy, clinical, laboratory, and patient-reported domains.

### Statistical analysis

All 36 variables were analyzed in relation to capillaroscopic pattern severity, both as ordinal and binary variables. Descriptive statistics were reported as medians and interquartile ranges (IQRs) for continuous variables, and as frequencies (percentages) for categorical variables.

- Ordinal associations between NVC patterns (2–5) and continuous variables were assessed using Spearman's rank correlation coefficient (ρ).
- For between-group comparisons, Mann–Whitney *U*-tests were used for continuous variables and Fisher's exact test for categorical comparisons (e.g., NVC patterns 2–3 vs. 4–5).
- Effect sizes (*r*) for Mann–Whitney *U*-tests were calculated to estimate the magnitude of differences between groups, based on the standardized z statistic and total sample size, in line with established non-parametric effect size estimation methods.
- Logistic regression models were constructed to assess the association between NVC pattern severity and ILD presence (Cutolo 4–5 vs. 2–3). An initial univariate model was followed by multivariable logistic regression analyses adjusting for clinically relevant covariates, including anti-Scl-70 status, disease duration, skin fibrosis (mRSS), and disease subtype (dcSSc vs. lcSSc). To minimize the risk of overfitting in this modest-sized cohort, parsimonious two-predictor models were also fitted.
- Model performance was summarized using the odds ratio (OR) and corresponding 95% confidence interval (CI) for the effect of NVC pattern severity on ILD, with model calibration assessed using the Hosmer–Lemeshow goodness-of-fit test.
- To evaluate the discriminative ability of NVC pattern severity for ILD detection, receiver operating characteristic (ROC) curves were constructed for both ordinal (2–5) and binary (2–3 vs. 4–5) NVC variables, and the area under the curve (AUC) was calculated.

Statistical tests were two-tailed, with α set at 0.05 and *p*-values were corrected using the Benjamini–Hochberg false discovery rate (FDR) method.

All statistical analyses were performed using Python 3.10 with the pandas, scipy.stats, statsmodels, and scikit-learn libraries. No imputation was performed; all analyses were based on complete cases (23, 24).

### Patient and public involvement

Patients or members of the public were not involved in the design, conduct, reporting, or dissemination plans of this research.

## Results

### Cohort characteristics

A total of 70 patients with systemic sclerosis (SSc) were included in the study (Table 1). The majority were female (*n* = 58, 82.9%), and the median age was 55.0 years (IQR: 47.0–68.0). Median disease duration was 3.0 years (IQR: 1.0–9.0). Among patients with available data (*n* = 68), 36.8% had higher education, and 76.5% resided in urban areas. Fifty percent (*n* = 34) were employed, and 61.8% lived with children.

NVC patterns distribution included: pattern 2 (non-specific abnormalities) in 14.3%, pattern 3 (early) in 48.6%, pattern 4 (active) in 27.1%, and pattern 5 (late) in 10.0% of patients. Patterns 1 (normal) and 6 (scleroderma-like) were not observed. Based on binary groupings, 62.9% of patients fell within patterns 2–3 and 37.1% within patterns 4–5. Pattern 2 alone was present in 14.3% of patients, while SSc specific patterns 3–5 accounted for 85.7%.

PROMs revealed a median SScQoL score of 13.5 (IQR: 9.0–20.0), HAQ-DI of 0.8 (0.2–1.2), VAS pain of 50.0 mm (25.8–72.2), and PtGA of 51.5 mm (32.8–67.5).

ILD was present in 34.3% (24/69) of patients, PAH in 7.1%, and gastrointestinal involvement in 21.4%. The median mRSS was 5.0 (IQR: 2.0–8.5).

Among laboratory markers, ESR was 14.0 mm/h (8.0–26.0), CRP 1.9 mg/L (0.8–3.9), IL-6 3.8 pg/ml (2.1–6.9), creatinine 0.7 mg/dl (0.7–0.9), and eGFR 95.0 mL/min/1.73 m^2^ (83.0–107.0).

Autoantibody profiles showed ACA positivity in 51.5%, Scl-70 in 48.5%, and anti-Th/To antibodies in 28.6% (*n* = 18/63). Median complement C3c was 114.6 mg/dl (102.0–126.7), and C4 was 24.8 mg/dl (17.7–28.8).

Current or past exposure to immunosuppressive therapy included MMF in 42.9%, MTX in 41.4%, AZA in 4.3%, and CYC (historical use only, none in past 6 months) in 8.6%. Current use of intravenous iloprost was documented in 62.9% of patients.

### Association between NVC patterns and ILD

A significant association was observed between NVC pattern severity and the presence of ILD. Using Spearman rank correlation, ILD status positively correlated with ordinal NVC score (patterns 2–5), with a correlation coefficient of ρ = +0.334 (95% *CI*: 0.10–0.55, *p* = 0.0051; FDR-adjusted *q* = 0.0058). When NVC patterns were grouped into lower severity (patterns 2–3) vs. higher severity (patterns 4–5), ILD prevalence increased markedly: 22.7% in the 2–3 group vs. 56.0% in the 4–5 group (*r* = 0.34, *p* = 0.0058; *q* = 0.0058) (Table 2A).

**Table 2A:** Significant associations between NVC patterns and ILD.

Variable	*N*	ρ (Spearman)/%	95% *CI ρ*/*r*	*p*-value	FDR
1. Spearman correlation between NVC patterns (ordinal scale 2–5) and ILD					*q*-value
ILD	69	ρ = +0.334	0.10–0.55	0.0051	0.0058
2. Differences in ILD prevalence between NVC patterns 2–3 vs. 4–5 (Mann–Whitney)					*q*-value
ILD 2–3 vs. 4–5	44/25	22.7%/56%	*r* = 0.34	0.0058	0.0058

**Figure 1:**
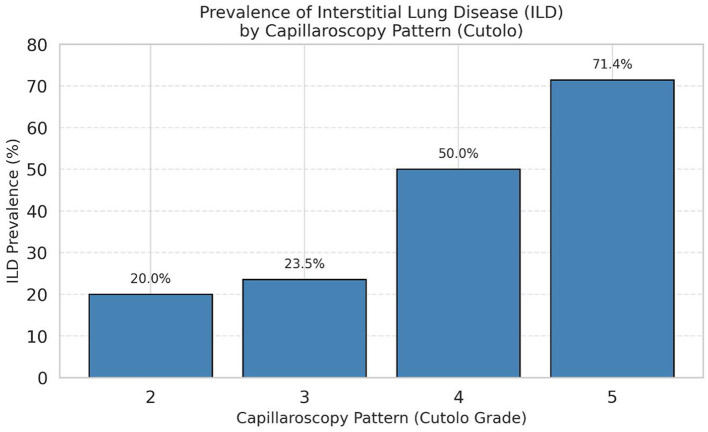
Prevalence of interstitial lung disease (ILD) across NVC pattern severity groups.

These findings were further supported by binary logistic regression, in which more advanced NVC patterns (4–5) were significantly associated with higher odds of ILD diagnosis [odds ratio (OR) = 3.73, 95% *CI*: 1.42–9.81, *q* = 0.0058] (Figure 1).

Logistic regression analysis confirmed that advanced NVC patterns (4–5) were significantly associated with the presence of ILD (*OR* = 3.73, 95% *CI*: 1.42–9.81, *p* = 0.0076, *q* = 0.0058), with a Nagelkerke *R*^2^ of 0.13 and adequate model calibration (Hosmer–Lemeshow *p* = 0.585).

**Figure 2:**
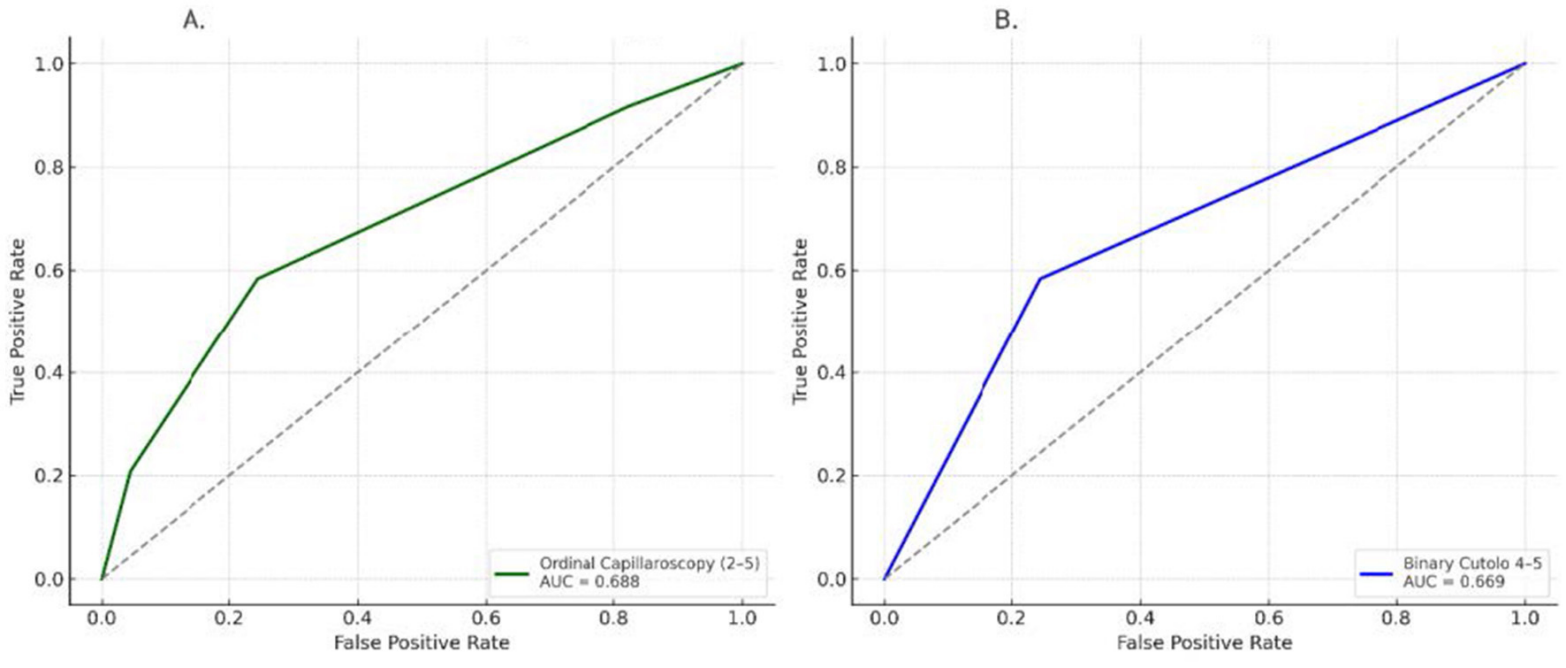
Receiver operating characteristic (ROC) curves assessing the diagnostic performance of NVC pattern severity for detecting interstitial lung disease (ILD). **(A)** ROC curve - ordinal capillaroscopy. **(B)** ROC curve - binary cutoio 4-5.

Discrimination analyses further demonstrated that both ordinal and binary NVC pattern variables had moderate ability to distinguish ILD status. The area under the ROC curve was 0.688 for the ordinal score (grades 2–5) and 0.669 for the binary grouping (2–3 vs. 4–5) (Figure 2).

To further examine whether the observed association between NVC severity and ILD persisted after adjustment for established clinical predictors, multivariable logistic regression models were constructed. Across five model specifications, advanced NVC patterns (4–5 vs. 2–3) were consistently associated with higher odds of ILD. In models adjusting for anti-Scl-70 status together with disease duration or skin fibrosis (mRSS), the odds ratios for NVC severity were 4.90 (95% *CI*: 1.49–16.18, *p* = 0.009) and 4.02 (95% *CI*: 1.26–12.82, *p* = 0.019), respectively. Parsimonious two-predictor models yielded similar effect estimates, with odds ratios of 4.27 (95% *CI*: 1.40–13.01, *p* = 0.011) and 4.49 (95% *CI*: 1.48–13.61, *p* = 0.008). In the model including anti-Scl-70 status and disease subtype (dcSSc vs. lcSSc), the association remained directionally consistent but did not reach statistical significance (*OR* = 3.17, 95% *CI:* 0.88–11.34, *p* = 0.076). All models were based on complete-case analysis. The number of ILD events allowed fitting models with acceptable events-per-variable ratios, ranging from 6.0 to 7.0 in three-predictor models and exceeding 10 in two-predictor models (Table 2B).

**Table 2B:** Multivariable logistic regression models assessing the association between NVC severity and ILD.

Variable	Model 1	Model 2	Model 3	Model 4	Model 5
	ILD ~NVC + Scl-70 + disease duration	ILD ~NVC + Scl-70 + mRSS	ILD ~NVC + Scl-70 + subtype	ILD ~NVC + Scl-70	ILD ~NVC + disease duration
NVC (patterns 4–5 vs. 2–3)	4.90 (1.49–16.18) *p* = 0.009	4.02 (1.26–12.82) *p* = 0.019	3.17 (0.88–11.34) *p* = 0.076	4.27 (1.40–13.01) *p* = 0.011	4.49 (1.48–13.61) *p* = 0.008
Anti-Scl-70 positivity	2.92 (0.89–9.63) *p* = 0.078	1.84 (0.57–6.00) *p* = 0.310	0.81 (0.06–10.85) *p* = 0.876	2.47 (0.81–7.55) *p* = 0.113	–
Disease duration (years)	1.08 (0.98–1.19) *p* = 0.104	–	–	–	1.05 (0.96–1.15) *p* = 0.289
mRSS	–	1.03 (0.94–1.14) *p* = 0.504	–	–	–
dcSSc vs. lcSSc subtype	–	–	6.40 (0.49–83.51) *p* = 0.157	–	–
Sample size (*N*)	65	63	59	66	68
ILD events	21	20	18	22	23
AUC	0.754	0.753	0.775	0.724	0.709
Nagelkerke *R*^2^	0.222	0.183	0.282	0.193	0.151
AIC	78.57	77.91	67.47	80.16	85.15
Hosmer–Lemeshow *p*	0.820	0.268	0.710	0.946	0.384

### Association between NVC patterns and skin fibrosis (mRSS)

Progressively advanced NVC patterns were significantly associated with more severe skin involvement, as measured by mRSS. On ordinal analysis, a moderate positive correlation was observed between NVC patterns severity (2–5) and mRSS (ρ = +0.376, 95% *CI*: 0.150–0.570, *p* = 0.0093, *q* = 0.0283).

When analyzed categorically, patients with advanced NVC changes (patterns 4–5) had higher skin scores than those with earlier-stage patterns (2–3), with a median mRSS of 7.0 vs. 4.0, respectively (*r* = 0.319, *q* = 0.0488). Similarly, patients with pattern 2 (non-specific microangiopathy) had lower mRSS values (median 2.0) than those with patterns 3–5 (median 6.0; *r* = 0.299, *q* = 0.0283) (Table 3).

**Table 3:** Significant associations between mRSS and NVC patterns.

Variable	*N*/groups	Statistic/median	Effect size/95% *CI*	*p*-value	FDR
Spearman correlation between mRSS and NVC patterns (ordinal scale 2–5)					*q*-value
mRSS	67	ρ = +0.376	95% *CI*: 0.150 – 0.570	0.0093	0.0283
Differences between NVC patterns (Mann–Whitney)					*q*-value
mRSS 2–3 vs. 4–5	41/26	4.0/7.0	*r* = 0.319	0.039	0.0488
mRSS 2 vs. 3–5	10/57	2.0/6.0	*r* = 0.299	0.017	0.0283
Differences in mRSS and NVC pattern between ACA+ and Scl70+ SSc patients					
Variable	*N* (ACA+/Scl70+)	Median (ACA+/Scl70+)	Effect size (*r*)	*p*-value	*q*-value
mRSS	34/32	3.0/9.0	0.315	0.0132	0.0283
NVC pattern	34/32	3.0/4.0	0.179	0.1462	0.1462

**Figure 3:**
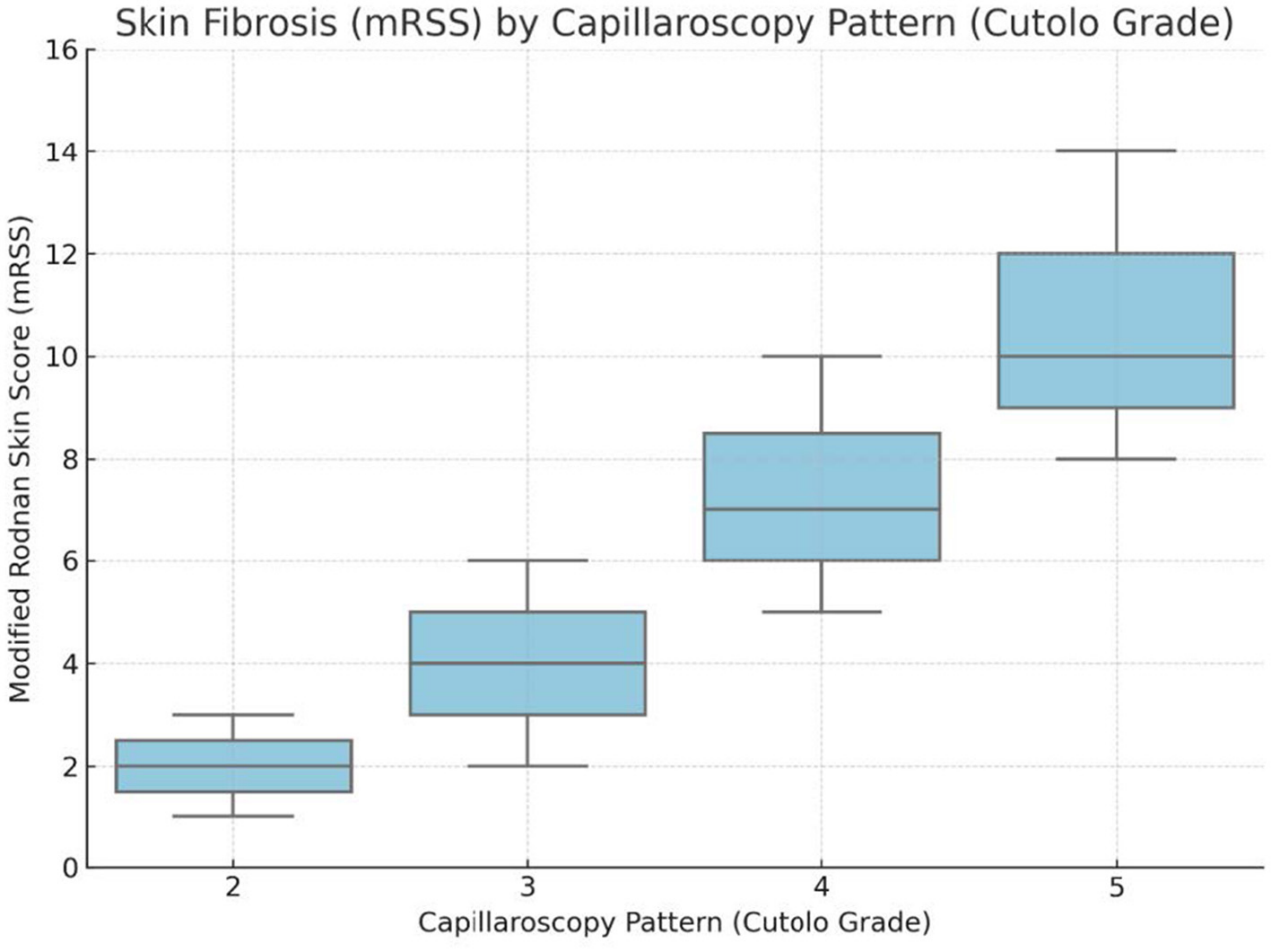
Association between NVC patterns and skin fibrosis severity.

These differences were visually supported by a monotonic increase in mRSS medians from 2.0 in pattern 2 to 10.0 in pattern 5, as illustrated in Figure 3.

In addition, when comparing major autoantibody-defined subsets, patients positive for anti-Scl-70 antibodies had markedly higher skin scores than ACA-positive individuals (median mRSS 9.0 vs. 3.0; *r* = 0.315, *q* = 0.0283). However, the corresponding NVC pattern severity (ordinal score) between these groups did not differ significantly (median score 4.0 vs. 3.0; *p* = 0.1462, *q* = 0.1462) (Table 3).

### Association between NVC patterns and SSc—specific autoantibodies

Significant serological correlations were observed between NVC pattern severity and SSc-specific autoantibodies. On ordinal analysis (NVC patterns 2–5), both anti-centromere antibody (ACA) and anti-Th/To antibody positivity were negatively correlated with NVC severity (ρ = −0.29 and −0.30, respectively; both *q* = 0.022), indicating lower prevalence of these antibodies in patients with more advanced microangiopathy.

Between-group comparison confirmed these findings: ACA was present in 64.3% of patients with mild NVC changes (2–3), compared to 33.3% in the more severe group (4–5; *q* = 0.022), with an effect size *r* = −0.31 (Table 4).

**Table 4:** Significant associations between NVC patterns and SSc-specific autoantibodies.

Variable	*N*	ρ (Spearman)/%	95% *CI ρ*/*r*	*p*-value	FDR
1. Spearman correlation between NVC patterns (ordinal scale 2–5) and ACA/Th/To					*q*-value
ACA	63	−0.29	−0.50 to −0.04	0.022	0.022
Th/To	63	−0.30	−0.51 to −0.06	0.015	0.022
2. Differences in ACA prevalence between NVC patterns 2–3 vs. 4–5 (Mann–Whitney)					*q*-value
ACA 2–3 vs. 4–5	42/21	64.3%/33.3%	−0.31	0.022	0.022

To better illustrate the distribution underlying the anti-Th/To association, capillaroscopic grades were examined within the antibody-positive subgroup. Among anti-Th/To–positive patients (*n* = 18), 6 had grade 2, 9 had grade 3, 2 had grade 4, and 1 had grade 5. Thus, 15 of 18 patients presented with grades 2–3, whereas 3 exhibited grades 4–5.

Among anti-Th/To–negative patients, 27 had grades 2–3 and 18 had grades 4–5. The corresponding 2 × 2 distribution (anti-Th/To positive vs. negative × NVC patterns 2–3 vs. 4–5) was therefore 15/3 vs. 27/18, respectively. Fisher's exact test confirmed the inverse association (p = 0.043), consistent with the observed inverse ordinal correlation (ρ = −0.30). The fragility index for this 2 × 2 association was 1.

### Association between NVC patterns and PROMs

Higher NVC pattern severity was significantly associated with worse patient-reported outcomes across three out of four domains assessed. A moderate positive correlation was observed between NVC pattern (ordinal scale 2–5) and SScQoL score (ρ = +0.285, 95% *CI*: 0.050–0.490, *p* = 0.019, *q* = 0.0040) as well as with HAQ-DI (ρ = +0.323, 95% *CI*: 0.092–0.522, *p* = 0.007, *q* = 0.0027).

When analyzed as a binary variable, patients with advanced NVC patterns (grades 4–5) had significantly higher median SScQoL scores (19.0 vs. 12.0; *r* = 0.35, *p* = 0.004, *q* = 0.0040), reflecting poorer health-related quality of life. Similarly, HAQ-DI values were elevated in the 4–5 NVC patterns group (median 1.19 vs. 0.625; *r* = 0.44, *p* < 0.001, *q* = 0.0027), indicating greater functional disability. The PtGA was also higher in patients with more severe NVC changes (median 65.0 vs. 45.0; *r* = 0.35, *p* = 0.004, *q* = 0.0040), suggesting greater perceived disease burden (Table 5).

**Table 5:** Significant associations between PROMs and NVC patterns.

PROM	*N*	ρ (Spearman)	95% *CI ρ*	*p*-value	*q*-value
Spearman correlation between PROMs and NVC patterns (ordinal scale 2–5)					FDR
SScQoL	68	+0.285	0.050–0.490	0.019	0.0040
HAQ-DI	68	+0.323	0.092–0.522	0.007	0.0027
PROM	*N* (2–3/4–5)	Median (2–3/4–5)	Effect size (*r*)	*p*-value	*q*-value
Differences between NVC patterns 2–3 vs. 4–5 (Mann–Whitney)					FDR
SScQoL	42/26	12.0/19.0	0.35	0.004	0.0040
HAQ-DI	42/26	0.625/1.19	0.44	< 0.001	0.0027
PtGA	42/26	45.0/65.0	0.35	0.004	0.0040

With the exception of PtGA, all statistically significant group comparisons across clinical, immunological, and patient-reported domains showed effect sizes in the range of *r* = 0.28–0.44, indicating robust between-group differences.

### Association between NVC patterns and immunosuppressive therapy

Advanced NVC patterns were significantly associated with exposure to selected immunosuppressive treatments. A moderate positive correlation was observed between NVC severity (ordinal scale 2–5) and both CYC past exposure (ρ = +0.30, 95% *CI*: 0.07–0.50, *p* = 0.011, *q* = 0.019) and MMF present or past exposure (ρ = +0.28, 95% *CI*: 0.05–0.48, *p* = 0.019, *q* = 0.019).

When compared categorically, patients with NVC patterns 4–5 were more likely to have received CYC (19.2%) than those with patterns 2–3 (2.3%; *r* = +0.17, *p* = 0.016, *q* = 0.019), reflecting a treatment pattern consistent with greater disease severity.

### Variables not significantly associated with NVC patterns

All study variables were evaluated for association with NVC patterns, both ordinally (patterns 2–5) and categorically (2–3 vs. 4–5). A subset showed no significant correlation or between-group difference after false discovery rate (FDR) correction and is summarized below to ensure completeness.

Demographic and Socioeconomic Variables

Sex, age, education level, place of residence, family status, and employment were not significantly associated with NVC severity in either analysis.

Clinical Features

No associations were observed with disease duration, PAH, or gastrointestinal involvement.

Inflammatory and Renal Biomarkers

ESR, CRP, and IL-6 showed no correlation with NVC severity. Similarly, creatinine and eGFR were unrelated to NVC patterns.

Immunological Parameters

Complement C3c, C4, and anti-Scl-70 antibody positivity were not significantly associated with NVC severity.

PROMs

SScQoL and HAQ-DI were significantly associated with NVC as described above. However, VAS pain showed no correlation or between-group difference. PtGA was elevated in advanced patterns (2–3 vs. 4–5), though not significantly correlated on an ordinal scale.

Treatment Variables

No significant associations were found for MTX, AZA, or ongoing iloprost therapy.

These negative findings provide important context for interpreting NVC patterns in systemic sclerosis.

## Discussion

This cross-sectional study systematically examined the relationship between NVC patterns and a broad spectrum of SSc manifestations, including internal organ involvement, skin fibrosis, autoantibody profiles, immunosuppressive therapy, and patient-reported outcomes. Using standardized NVC patterns classification, we identified consistent associations with ILD, mRSS, and anti-Th/To positivity, as well as strong correlations with disability and quality of life. The use of formal regression modeling, standardized scoring, and FDR correction addresses key limitations of prior studies and supports the robustness of our findings.

### Cohort characteristics and external validity

The study included 70 patients with systemic sclerosis, predominantly female (82.9%), with a median age of 55 years and a median disease duration of 3 years. The distribution of SSc-specific autoantibodies showed a comparable prevalence of ACA (51.5%) and anti-Scl70 (48.5%), consistent with proportions reported in the Spanish RESCLE cohort (43.5% and 39.6%, respectively) (25). Interstitial lung disease (ILD) was identified in 34.3% of patients, a frequency similar to that observed in other cohorts (25, 26).

This close alignment with large European cohorts such as EUSTAR (26) and RESCLE (25) supports the external validity of our findings and enhances the generalizability of observed associations between nailfold capillaroscopic (NVC) patterns and systemic outcomes. Unlike registry-based studies, our dataset included systematically collected PROMs, inflammatory markers, and capillaroscopic assessments using a standardized ordinal grading system, improving data precision and internal consistency.

### Nailfold microangiopathy and ILD

ILD was identified in 24 of 69 patients (34.3%), consistent with EUSTAR and RESCLE registry data (25, 26). ILD-positive and ILD-negative subgroups did not differ significantly in age, sex, or disease duration, but ILD was more frequent in patients with diffuse cutaneous SSc and anti-Scl70 antibodies (Table 1). Over 50% of ILD patients received mycophenolate or cyclophosphamide, compared to 33% in ILD-negative individuals (27).

Despite immunosuppression, ILD patients showed more severe capillaroscopic abnormalities. Ordinal NVC patterns (grades 2–5) were significantly associated with ILD (*p* = 0.011, *q* = 0.022), with late NVC patterns (4–5) conferring a 3.73-fold increased ILD risk (95% *CI*: 1.42–9.81) relative to milder cases (Table 2A). Importantly, the initial logistic regression model was unadjusted; therefore, independence from established ILD predictors could not be inferred. Multivariable logistic regression analyses adjusting for key clinical predictors, including anti-Scl-70 status, disease duration, and skin fibrosis (mRSS), confirmed that advanced NVC patterns remained significantly associated with ILD, with odds ratios of approximately four across model specifications (Table 2B). Taken together, these findings indicate that the association between NVC severity and ILD remains consistent across several adjusted model specifications, with effect estimates around a fourfold increase in the odds of ILD. To our knowledge, this study is the first to demonstrate this association using adjusted multivariable models in systemic sclerosis. ROC analysis demonstrated modest discriminatory performance for both ordinal (AUC = 0.688) and binary (AUC = 0.669) classifications (Figures 1, 2). An AUC of 0.688 indicates that NVC severity correctly ranks an ILD-positive patient above an ILD-negative patient in approximately 69% of cases, suggesting that capillaroscopy may serve as an adjunctive screening marker rather than a standalone diagnostic tool.

These results are consistent with earlier studies from Italy and India (28, 29), but differ methodologically through the use of prospectively graded NVC, HRCT-confirmed ILD, and FDR-adjusted statistics (30, 31). The moderate effect size (*r* = 0.34) suggests a clinically meaningful difference in microvascular involvement between ILD and non-ILD patients.

Late NVC patterns (capillary dropout, avascularity) may reflect systemic microangiopathy occurring alongside fibrotic lung involvement. Supporting evidence includes elevated epithelial injury markers (KL-6, CA 15-3) in patients with concurrent NVC damage and ILD (21, 32). Our previous work demonstrated that CA 15-3 is significantly associated with ILD and linked to anti-PM/Scl antibodies (32).

Persistent vascular–pulmonary associations despite therapy may indicate that microangiopathy and fibrotic lung involvement follow partially independent pathways. Similar findings were reported in the SENSCIS substudy (33).

Our results are consistent with observations from the SCLEROCAP cohort, where baseline NVC scores were associated with the subsequent development of major organ complications, including ILD (34). While cross-sectional, the reproducibility of associations across methods and cohorts supports their clinical relevance.

Advanced NVC abnormalities may reflect a systemic vascular phenotype and support the use of NVC as a non-invasive marker associated with ILD status, particularly where HRCT or biomarkers such as KL-6 are inaccessible.

### Skin fibrosis (mRSS) and microangiopathy patterns

A moderate correlation was observed between NVC severity and skin fibrosis, as measured by mRSS (ρ = +0.376, *q* = 0.0283) (Table 3). Median mRSS increased stepwise across NVC categories: from 2.0 (pattern 2) to 4.0 (pattern 3), 7.0 (pattern 4), and 10.0 (pattern 5) (Figure 3), distribution of microvascular and fibrotic damage across NVC categories.

Patients with late NVC patterns (4–5) had significantly higher mRSS than those with earlier patterns (2–3) (median 7.0 vs. 4.0; *r* = 0.319, *q* = 0.0488). A similar difference was noted between “non-specific” (2) and SSc-specific (3–5) patterns (2.0 vs. 6.0; *r* = 0.299, *q* = 0.0283), reinforcing the concept of cumulative fibrotic burden with advancing microangiopathy.

These results extend earlier findings by Ingegnoli et al. (26) and Zumstein Camargo et al. (35) by incorporating FDR correction and stratified analyses across NVC categories.

Serological profiles mirrored these associations. Scl70-positive patients exhibited higher mRSS than ACA-positive individuals (9.0 vs. 3.0; *r* = 0.315, *q* = 0.0283). Although the NVC score difference between these groups was not statistically significant (median 4.0 vs. 3.0, *q* = 0.1462), the trend aligns with more severe disease in Scl70+ patients. Conversely, ACA positivity correlated with lower mRSS and milder NVC patterns, consistent with prior studies (30, 31).

These findings support the value of NVC as a vascular correlate of skin fibrosis in SSc. While causality cannot be inferred from cross-sectional data, the coherence across scales and subgroups suggests a shared pathophysiological trajectory. Longitudinal studies are needed to clarify the temporal relationship between capillaroscopic and fibrotic progression.

### Autoantibodies and microangiopathy patterns

We identified significant associations between NVC severity and specific SSc-related autoantibodies. Patients with advanced microangiopathy (NVC patterns 4–5) had a markedly lower prevalence of ACA compared to those with milder patterns (2–3; 33.3% vs. 64.3%, *q* = 0.022; Table 4), with an inverse correlation between NVC severity and ACA positivity (ρ = −0.29, *q* = 0.022). This finding is consistent with extensive prior evidence showing that ACA-positive patients tend to exhibit limited cutaneous disease and relatively preserved microvascular architecture (36). Large datasets such as the EUSTAR capillaroscopy registry confirm this association, with ACA linked to early or active NVC patterns, and anti-Scl-70 to more advanced microangiopathy (26, 30, 37).

Importantly, our study is the first to report a statistically significant inverse correlation between anti-Th/To antibodies and capillaroscopic severity (ρ = −0.30, *q* = 0.022) using standardized NVC grading. Within the anti-Th/To–positive subgroup (*n* = 18), 15 patients exhibited NVC patterns 2–3 and 3 presented patterns 4–5, directly reflecting the distribution underlying the reported inverse correlation. However, the number of anti-Th/To–positive patients with advanced NVC patterns was small (3 cases), and the observed association should therefore be interpreted cautiously and considered hypothesis-generating pending confirmation in larger cohorts. While previous studies have linked Th/To antibodies to increased ILD risk and adverse pulmonary outcomes (38–41), no prior work has formally evaluated their association with peripheral microvascular damage. The present finding introduces novel evidence that Th/To-positive patients may display relatively preserved capillaroscopic architecture despite systemic fibrotic risk, suggesting a dissociation between pulmonary and peripheral vascular compartments in this serological subset. This contributes to emerging efforts to refine SSc endotyping by integrating serology and vascular phenotype.

In contrast, no statistically significant difference in NVC scores was found between Scl70- and ACA-positive individuals (median NVC 4.0 vs. 3.0, *q* = 0.1462), although skin scores were significantly higher in Scl70-positive patients (median mRSS 9.0 vs. 3.0, *r* = 0.315, *q* = 0.0283). This pattern aligns with previous reports linking Scl70 to more severe cutaneous involvement and progressive fibrosis (7, 27). The lack of significant association between anti-Scl-70 positivity and NVC severity in our cohort may reflect cohort-specific characteristics and the application of multiple-testing correction, rather than a deterministic autoantibody–capillaroscopy relationship.

Taken together, our results reaffirm the role of SSc-specific autoantibodies not only as diagnostic markers but also as correlates of microvascular involvement. While the ACA-related findings strengthen external validity through consistency with prior datasets, the newly described anti-Th/To association provides original insight into capillaroscopic stratification in a poorly characterized serological subset.

### Functional impact and PROMs

A key novel finding of this study is the robust association between capillaroscopic severity and PROMs, underscoring the functional relevance of microangiopathy in SSc. Patients with advanced NVC patterns (4–5) reported significantly worse outcomes across all PROMs: higher SScQoL (19.0 vs. 12.0, *r* = 0.35, *q* = 0.0040), HAQ-DI (1.19 vs. 0.625, *r* = 0.44, *q* = 0.0027), and PtGA scores (65.0 vs. 45.0, *r* = 0.35, *q* = 0.0040) (Table 5). Correlations between NVC severity and PROMs remained moderate but statistically significant (ρ = +0.285 to +0.323, all *q* < 0.005), suggesting a graded relationship between microvascular damage and patient-perceived burden.

To our knowledge, this is the first study to report such associations using validated, SSc-specific and non-specific PROMs (SScQoL, HAQ-DI, PtGA) alongside ordinal NVC grading and multiple testing correction. Prior studies linked PROMs to disease burden and NVC to organ involvement (13, 14, 28), but none demonstrated direct links between capillaroscopic severity and quality of life.

These findings suggest that microangiopathy may be associated with disability and reduced quality of life in SSc, potentially through mechanisms such as Raynaud's severity, stiffness, or reduced perfusion—even in the absence of overt organ involvement. This is supported by experimental data associating avascular areas with digital ischemia and musculoskeletal symptoms (27).

The associations remained detectable despite the cohort's moderate disease burden (median HAQ-DI 0.8; SScQoL 14.0), relatively short disease duration (3.0 years), and predominance of lcSSc, emphasizing their relevance even in early-stage disease.

Methodologically, use of ordinal NVC patterns (2–5) enhanced resolution of vascular injury over binary staging. In contrast to earlier studies with small samples and uncorrected *p*-values, our use of FDR-adjusted statistics and disease-specific PROMs increases reproducibility and interpretability (14).

Interestingly, VAS pain scores did not correlate with NVC severity, supporting the idea that SSc pain arises from mechanisms distinct from microangiopathy, such as small fiber neuropathy or musculoskeletal damage (11).

Altogether, these data support the role of capillaroscopy as a potential correlate of functional status in SSc, with implications for monitoring and trial design. However, given the cross-sectional design, these associations should not be interpreted as evidence of a direct causal effect of microvascular damage on quality of life, as impaired patient-reported outcomes may also reflect internal organ involvement and overall disease severity co-occurring with advanced NVC patterns. Future studies should explore whether interventions targeting microvascular damage yield measurable PROM benefits.

### Treatment associations with NVC severity

In our cohort, advanced NVC patterns (4–5) were significantly associated with prior exposure to CYC (19.2% vs. 2.3%, *q* = 0.019), and showed positive correlations with both CYC (ρ = +0.30, *q* = 0.019) and MMF (ρ = +0.28, *q* = 0.019). These associations likely reflect clinical escalation of immunosuppression in patients with severe phenotypes, including ILD and diffuse cutaneous involvement, which commonly co-occur with advanced microangiopathy.

These findings align with the multicenter RESCLE study (25), which similarly reported increased use of MMF and CYC in patients with progressive NVC patterns and systemic complications. This cross-cohort concordance strengthens the external validity of our results and suggests that NVC may indirectly inform treatment intensity in practice.

In contrast, no significant associations were found for MTX or AZA. Similarly, iloprost use did not differ between NVC severity groups, reinforcing prior RESCLE data showing no effect of vasodilator therapy on capillaroscopic progression (25).

Overall, these findings should be interpreted as reflecting treatment patterns driven by systemic disease severity captured by NVC, rather than any direct pharmacological effect on capillaroscopic morphology. The cross-sectional design precludes causal inference.

### Absence of associations with inflammatory, renal, and serological markers

While strong correlations were observed between NVC severity and above mentioned domains, other parameters showed no meaningful relationship with NVC patterns. These negative results help delineate the interpretive scope of NVC as a vascular biomarker in SSc.

Systemic inflammatory markers—CRP, ESR, and IL-6—were not significantly correlated with NVC scores. This suggests that peripheral microangiopathy progresses largely independently of systemic inflammation, supporting prior findings that CRP and ESR are poor indicators of vascular injury in SSc (18, 21). These results reinforce the concept of NVC changes as reflecting chronic structural remodeling rather than dynamic inflammatory activity.

Similarly, no associations were found between NVC severity and renal function markers. This is consistent with the distinct pathophysiology of scleroderma renal involvement (27).

Finally, immunoglobulin levels (IgG, IgM) and complement components (C3c, C4) did not vary significantly across NVC strata.

Collectively, these findings suggest that NVC severity does not reflect global disease burden or humoral immune activation, but rather represents a distinct microvascular phenotype.

### Limitations and strengths

This study has several limitations.

First, its cross-sectional design precludes causal inference and temporal assessment of capillaroscopic progression relative to organ involvement. Although robust associations were identified, it remains unclear whether microangiopathy precedes, parallels, or follows domains such as ILD or fibrosis.

Additionally, even though age was recorded, detailed data on smoking status, hypertension, and other vascular comorbidities were not systematically available for all participants; therefore, residual confounding by these factors cannot be excluded.

Moreover, ILD was analyzed as a binary HRCT-confirmed variable; quantitative assessment of radiological extent, HRCT subtypes, and pulmonary function parameters (e.g., FVC, DLCO) was beyond the scope of the predefined study design.

Associations with immunosuppressive therapies (CYC, MMF) may be influenced by confounding by indication; dose, duration, and cumulative exposure were not evaluated within the predefined study design.

Although the applied EULAR/Cutolo NVC classification integrates semi-quantitative parameters such as capillary density, giant capillaries, hemorrhages, avascular areas, and neoangiogenesis, individual modeling of these components was beyond the predefined scope of the present study. Future investigations focusing on detailed morphometric analysis may further elucidate which specific microvascular features drive organ-specific associations.

Second, the modest sample size (*n* = 70) may have limited statistical power, especially for rare NVC patterns (2 and 5). This necessitated binary grouping (2–3 vs. 4–5), improving analytical stability but potentially masking distinctions between individual patterns.

Third, the single-center, inpatient-based design may limit generalizability to fully unselected outpatient SSc populations but also conferred important methodological strengths. All patients were evaluated using harmonized protocols, and NVC was performed and interpreted by one EULAR-certified physician with dual training in rheumatology and dermatology. This minimized inter-observer variability and enhanced internal validity—a common challenge in multicenter cohorts.

Fourth, while false discovery rate (FDR) correction was applied, the number of comparisons performed still carries a risk of residual statistical noise. Novel associations, particularly those involving anti-Th/To antibodies or PROMs, should be considered exploratory and require external validation.

Finally, subgroup and sensitivity analyses were constrained by sample size, limiting interpretability in rare phenotypes such as late NVC pattern 5.

Despite these limitations, the study has notable strengths. The cohort closely mirrors larger registries such as EUSTAR and RESCLE in demographic and serological composition, supporting external validity. Standardized ordinal NVC scoring, rigorous statistical methodology, and integration of validated PROMs enhance granularity and reproducibility. The alignment of our findings with external datasets further reinforces their clinical relevance. Future studies in larger, ethnically diverse cohorts will be needed to assess generalizability and clarify longitudinal dynamics of microangiopathy in SSc.

## Conclusions

This study provides novel, comprehensive evidence linking nailfold microangiopathy severity to key systemic sclerosis domains—fibrotic, serological, immunological, therapeutic, and patient-reported. Using standardized ordinal NVC grading with FDR correction, we identified significant associations with ILD, skin fibrosis, anti-Th/To antibodies, immunosuppressive exposure, and multiple PROMs. To our knowledge, this is the first study integrating high-resolution capillaroscopic staging with validated, disease-specific PROMs. The consistency of our findings with external cohorts supports the validity of NVC as a reproducible, multidimensional biomarker, underscoring its potential role in SSc phenotyping, monitoring, and clinical trial design.

## Data Availability

The raw data supporting the conclusions of this article will be made available by the authors, without undue reservation.
